# Adherence to antiretroviral therapy among HIV patients in Ghana: A systematic review and meta-analysis

**DOI:** 10.1371/journal.pgph.0002448

**Published:** 2023-11-01

**Authors:** Isaac Boadu, Adom Manu, Richmond Nii Okai Aryeetey, Kwame Adjei Kesse, Marijanatu Abdulai, Emmanuel Acheampong, Robert Akparibo

**Affiliations:** 1 Department of Population, Family and Reproductive Health, School of Public Health, Legon, University of Ghana, Accra, Ghana; 2 Department of Epidemiology and Disease Control, School of Public Health, Legon, University of Ghana, Accra, Ghana; 3 Ghana National AIDS/STI Control Programme (NACP), Accra, Ghana; 4 Institute of Precision Health, University of Leicester, Leicester, United Kingdom; 5 School of Health and Related Research (ScHARR), University of Sheffield, Sheffield, United Kingdom; University of Embu, KENYA

## Abstract

Maintaining a high level of adherence to antiretroviral therapy (ART) is critical to limiting rapid viral replication, drug resistance, and viral transmission. However, ART adherence remains a major challenge in HIV/AIDS treatment success. This systematic review and meta-analysis was aimed to synthesize available evidence on adherence to ART among HIV/AIDS patients in Ghana.This review followed the preferred reporting item for systematic review and meta-analysis (PRISMA) criteria. A comprehensive literature search was done using five online databases (PubMed, Google Scholar, Medline, Africa Index Medicus, and Willey Online Library) from 25th- 30th April 2023 to identify potential studies. In addition, references of related articles were manually searched to further identify relevant studies. Search records were managed in Endnote library where duplicates were removed prior to screening. Studies were eligible for inclusion if they were conducted in Ghana, designed as an observational or experimental study, and explicitly measured adherence to ART, either as a primary or secondary outcome. Studies were excluded if the proportion or prevalence of adherence to ART was not reported.A total number of 126 potential studies were identified from the literature search. Of these, 14 met the inclusion criteria and were included in the Meta-analysis. The studies involved a total number of 4,436 participants. The pooled estimate of adherence to ART was 70% (CI: 58–81%). In subgroup analysis, adolescents and young adults had a lower adherence rate (66%, CI: 46–84%) compared with adults (70%; CI: 58–81%). Publication bias was not observed among studies. The pooled estimate of optimal adherence to ART among HIV patients in Ghana was lower than is recommended (≥95%) to achieve viral suppression. Adherence was lower among young persons living with HIV/AIDS. To achieve the United Nation’s Sustainable development goals and the UNAIDS “95-95-95” targets, there is a need to focus on improving adherence interventions among persons living with HIV/AIDS, especially among the younger cohort.

## Introduction

HIV/AIDS remains a significant global public health problem, having claimed more than 40 million lives, since its emergence over 40 years ago [1]. Globally, an estimated 38 million people were living with HIV (PLHIV) at the end of 2021 with 1.5 million new infections and about 650 thousand HIV-related deaths [1,2]. The HIV/AIDS burden is having a disproportionally higher impact on sub-Saharan Africa, where over two-thirds of all people living with HIV reside [2,3].

In Ghana, there has been modest progress in managing HIV/AIDS. In 2020, the national prevalence of HIV among adults in Ghana was estimated at 1.68% compared to 2.5% in 2002 including 22, 611 new infections [4].

The number of PLHIV, globally, continues to increase due to the effectiveness of ART in increasing survival and improving quality of life [5]. Early initiation of ART has been linked with reduced mortality and morbidity among PLHIV [5,6]. As a result, ART is recommended for all PLHIV, including children and adolescents. PLHIV should be put on ART as soon as possible, irrespective of the number of viral load [7].

In 2016, Ghana adopted and is implementing nationwide, the WHO’s ‘TREAT ALL’ policy on the use of ART for the treatment and prevention of HIV infection [4,8]. As a result, ART treatment coverage has improved significantly; coverage was estimated at 96% in 2020 [4]. Although Ghana is striving to meet national and global targets for treatment, the overall achievement of increasing testing, reducing new infections and mortality has been minimal. HIV-related deaths was estimated to be 13,616 in 2020 and 79% of PLHIV knew their serostatus at the end of 2022 [4].

Medication adherence is defined as the taking of all medications at the appropriate time and dosage as recommended by a physician [9,10]. Optimal adherence is important to ensure the efficacy of ART (achieve viral suppression), prevent drug resistance, lower opportunistic infections, reduce disease transmission, hospitality, morbidity and mortality, and ensure the well-being of PLHIV [11].

In the literature, several factors have been associated with adherence to ART. These include patient behavior and lifestyle factors (eg. forgetfulness, being busy, poor understanding of the regimen, self-stigma, etc.), treatment-related factors (side effects, polypharmacy), healthcare or provider factors (eg. waiting time, the attitude of health care providers) and socio-cultural factors (eg. use of traditional medicine, and spiritual beliefs) [12–17].

With a focus on achieving viral suppression, the joint United Nations Programme on HIV/AIDS (UNAIDS) launched the 95-95-95 initiative in 2020. This initiative seeks to achieve viral suppression among 95% of persons on ART [18]. Because adherence serves as a proxy for viral suppression [19], there is a need for focused attention to achieve this ambitious target.

Medication adherence of at least 95% is recommended as effective in achieving treatment success [20]. However, adherence to ART is low in sub-Saharan African countries (77%) [21]. For example, a systematic review in Ethiopia on adherence to highly active ART among children reported 88.7% of patients adhering to treatment on a 7-day recall [22]. In Ghana, several empirical studies have reported sub-optimal adherence among diverse HIV populations [23–30]. Biney et al. [26] reported an adherence level of 78.7% among adolescents and young adults in the Greater Accra region. Similarly, Obirikorang et al. [28] Sefah et al. [31] and Adu et al. [24] have reported adherence levels of 62.2, 42.9, and 53.1% among PLHIV, respectively. There is paucity of evidence on pooled adherence to ART to better understand HIV program success. This review, therefore, aims to report on pooled adherence rate of ART among persons living with HIV in Ghana and report on age-group adherence proportions. The review sought to address the following research questions: 1). What is the pooled adherence to ART among persons living with HIV/AIDS in Ghana?. 2). Are there differences in adherence level to ART among adults and young people living with HIV/AIDS in Ghana?.

## Methods

### Ethics statement

The study required no ethical approval.

### Search strategy

This review was registered in PROSPERO (Reg ID: CRD42023417543), and conducted following the preferred reporting item for systematic review and Meta-Analyses (PRISMA) guidelines [32]. A protocol for this review was prepared prior to the manuscript development. An extensive literature search was performed in Medline, PubMed, Africa Index Medicus (AIM), Google Scholar, and Wiley Online Library. We searched for published papers using the combination of free texts and medical subject headings (MeSH) terms, e.g. “Adherence” “non-adherence” “Compliance” “non-compliance” “ART” “HAART” “Highly active” “Antiretroviral therapy” “HIV/AIDS” and “Ghana”. We used the PICO framework for systematic reviews to structure the search using Boolean operator combinations. Supplemental searches were carried out by reviewing the references of the included papers, as well as follow-up searches of citations to identify additional papers that reported on medication adherence in Ghana but which were not identified through the electronic database searches. All searches were conducted from April 25 to 30, 2023.

### Eligibility criteria and selection criteria

Studies were included if (1) they were conducted in Ghana (2) used observational or experimental designs that explicitly measured adherence to ART, either as a primary or secondary outcome; For experimental studies, baseline adherence was considered; (3) mixed method studies that reported on the prevalence of adherence were also included. Consistent with a similar review [33], where more than one method was used to measure adherence, the more objective method used was reported. To give a wider scope of studies included, we did not limit the year of publication nor the age of study participants; we included published dissertations that met the inclusion criteria. Studies were excluded if they did not indicate evidence of prevalence of adherence to ART or did not meet the inclusion criteria 1, 2, and 3 described above.

**Fig 1:**
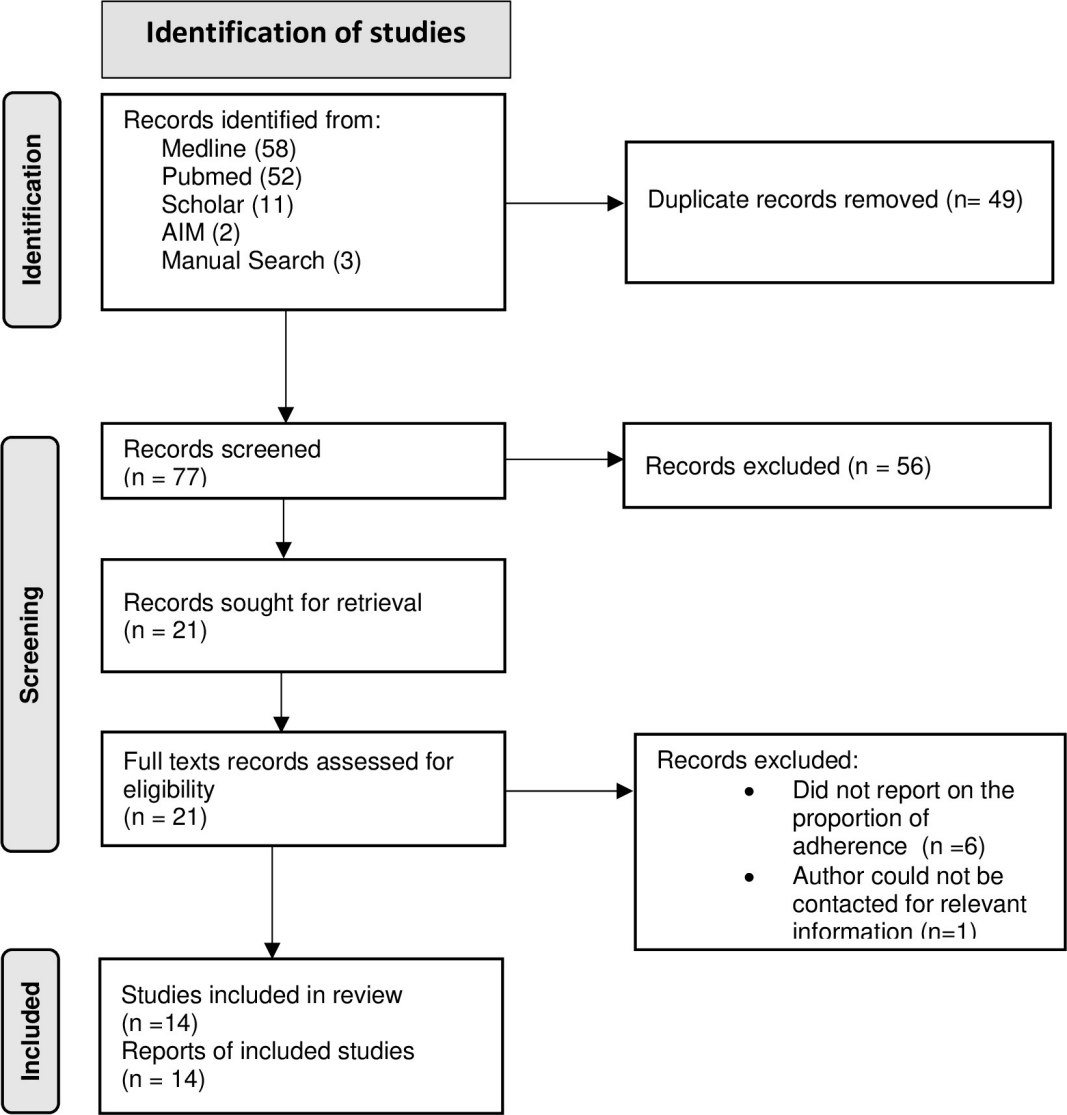
Flow chart showing study selection process [35]. For more information, visit: http://www.prisma-statement.org/.

All citations of identified documents were imported into EndNote 13 reference manager [34] and screened for relevance. Screening was carried out at three levels: title, abstract, and full text. At title and abstract levels, three reviewers (IB, MA, and KKA) independently applied a predetermined inclusion criteria to screen all titles and abstracts of the identified studies after duplicates were removed. Full texts of the potentially eligible abstracts were downloaded, read, and subsequently screened to ascertain their relevance with respect to the inclusion criteria. To eliminate any selection bias, a second reviewer (EA) screened 10% each of the included and excluded abstract and full texts. Any discrepancies were addressed between reviewers. The PRISMA flow chart (Fig 1) provides a summary description of the screening and selection process.

### Data extraction

Both text and statistical data were extracted from each of the articles meeting the inclusion criteria by two reviewers (IB and KKA). A third reviewer (MA) cross-checked the extraction for quality. Key information extracted included author detail, year of publication, study setting, study design, sample size, sampling method, the method used to measure adherence and proportion of adherence to ART. The authors of two studies [28,36] were contacted for additional relevant information we missed during the data extraction process.

### Quality assessment

Quality assessment of the included studies was done using the revised Joanna Briggs Institute (JBI) Critical Appraisal tools for systematic reviews [37]. This appraisal tool assesses the methodological quality of a study and determines the extent to which the study has addressed the possibility of bias in its design, data collection, and analysis [37]. Two authors (KKA and IB) carried out the quality assessment independently and graded the studies as low (>70%), medium (50–70%), and high (<50%) [37,38]. Discrepancies in the assessment and scoring were resolved through discussion and consensus.

### Statistical analysis

The extracted data were imported into R statistical software for analysis. Freeman-Tukey double arcsine transformation and inverse variance methods were used to calculate the pooled estimates of adherence to ART. The weighted random effects model was considered rather than the common effects due to the large statistical heterogeneity across studies [39–41]. As asserted by Fletcher [41], significant large heterogeneity can be handled in two ways; narrative synthesis or meta-analysis using random effects. The latter was used consistent with earlier studies [42,43] as included studies essentially measured the same outcome, that is, adherence to ART.

Documents were categorized by research participant’s age group: ‘adolescents and young adults’ (10–24 years) versus ‘adults’ (18+). This classification was based on WHO’s classification of young people [44]. Where there is age overlap, we used the reported mean to classify a study under these categories. Thus, when the reported mean age is greater than 24 years, the study was classified as an adult study or otherwise. We used the Chi^2^ Q test and I^2^ statistic to identify heterogeneity; an I^2^ estimate greater than 50% indicated significant heterogeneity [45]. Potential sources of heterogeneity were assessed using sensitivity and subgroup analyses. We performed exploratory analyses of heterogeneity using meta-regression based on the sample size of the studies and the quality scores of individual studies. Potential publication bias was evaluated using Egger’s and Begg’s tests. The R software package ‘*metafor’* was used to perform the analysis. All statistical analyses were 2-sided and statistical significance was considered at p-value < 0.05.

## Results

### Search outcome and study characteristics

A total of 126 records were retrieved from the databases and supplementary searches. Of these, 49 duplicates were identified and removed. We excluded 56 records after screening titles and abstracts. Twenty-one full-texts were assessed for eligibility, and 14 met our inclusion criteria and were subjected to review. These 14 studies were conducted between 2013 and 2022. The PRISMA flowchart shows the document selection process [Fig 1].

Shown in Table 1 are the characteristics of the 14 studies included in the meta-analyses. A total of 4,436 HIV-positive patients were studied across the 14 studies to ascertain their adherence to ART with sample sizes ranging from 25 [36] to 683 [30]. Diverse measurement methods were used to assess adherence to ART. These included studies that used self-reported questionnaires [24–26,28,30,46–48], pill count [31,36], Patient Attendance-based Defaulting (PAD) [49], and a combination of other methods [27,50] to determine adherence. Study designs used include surveys [23–26,28,30,31,36,47–49], randomized control trials [27,50], and a prospective cohort [46]. The studies were predominantly conducted in health facilities in the Ashanti (4/14; 28.6%) [24,25,46,49] and Greater Accra (4/14; 28.6%) [23,26,36,50] regions of Ghana. Other regions included Central [47], Eastern [31], Upper West [28]; a few were conducted in multiple regions [27,30,48]. Of the 14 studies, 10 (71.4%) were conducted among adults while 4 (28.6%) were among adolescents and young adults.

**Table 1:** Characteristics of the included studies on adherence to antiretroviral therapy in Ghana.

Author (s)	Year	Study Title	Study Location	Study design	Study Population	Sample size	Sampling method	Adherence Assessment	Prevalence Adherence
Biney et.al [26]	2021	Antiretroviral therapy adherence and viral suppression among HIV-infected adolescents and young adults at a tertiary hospital in Ghana	Greater Accra	Cross-sectional	Adolescents and young adults	136	Purposive	MMAS-8	78.67
Anokye-Kumatia et al. [25]	2018	Highly active antiretroviral therapy adherence among perinatally infected HIV adolescents at a teaching hospital in Ghana	Ashanti region	Cross-sectional	Adolescents and young adults	106	Random	MMAS-8	76.4
Addo et.al [23]	2022	Factors influencing adherence to antiretroviral therapy among HIV/AIDS patients in the Ga West Municipality, Ghana	Greater Accra	Cross-sectional	Adults	397	Purposive and systematic random	Self-report question	44.6
Nichols et al. [27]	2019	High prevalence of non-adherence to antiretroviral therapy among undisclosed HIV-infected children in Ghana	Ashanti region/ Greater Accra	Randomized clinical trial	children and adolescents	440	Random	Pharmacy refill, caregiver or child 3-day recall	47.5
Sefah et al. [31]	2022	Barriers and facilitators of adherence to antiretroviral treatment at a public health facility in Ghana: a mixed method study	Eastern region	Cross-sectional	Adults	231	Random	Pill count	42.9
Prah et al. [47]	2018	Factors affecting adherence to antiretroviral therapy among HIV/AIDS patients in Cape Coast Metropolis, Ghana	Central region	Cross-sectional	Adults	381	Systematic	Self-report (Life time adherence question)	73
Obirikorang et al. [28]	2013	Predictors of Adherence to Antiretroviral Therapy among HIV/AIDS Patients in the Upper West Region of Ghana	Upper west region	Cross-sectional	Adults	201	Convenience	Life time adherence question	62.2
Adu et al. [24]	2022	Socio-demographic factors associated with medication adherence among People Living with HIV in the Kumasi Metropolis, Ghana	Ashanti region	Cross-sectional	Adults	420	simple random	MMAS-8	53.1
Afrane et al. [36]	2021	HIV virological non-suppression and its associated factors in children on antiretroviral therapy at a major treatment centre in Southern Ghana: a cross-sectional study	Greater Accra	Cross-sectional	children and adolescents	25	consecutive	Pill count	61.6
Boateng and Kumah [49]	2021	Assessing the rate of antiretroviral therapy adherence among people living with HIV/aids in the Atwima Nwabiagya Municipal—Ashanti region	Ashanti region	Cross-sectional	adults	450	convenience	Patient Attendance-based Defaulting (PAD)	47.8
Adam et al. [48]	2022	Impact of antiretroviral therapy regimens adherence on perceived health and wellbeing status among adults living with HIV in Ghana	Volta and Oti Region	Cross-sectional	Adults	301	convenience	Self-report (Life time adherence question)	97.3
Annison et al. [46]	2013	The Immunological response of HIV-Positive patients initiating HAART at the Komfo Anokye Teaching Hospital, Kumasi, Ghana.	Ashanti region	follow up (prospective)	Adults	303	convenience	self-report (no treatment interruption)	86.5
Dzansi [50]	2017	Integrated mobile phone interventions for adherence to Antiretroviral treatment in clients with HIV infection in Accra, Ghana	Greater Accra	Randomized clinical trial	Adults	362	Random	Self-report, Pill count, VAS	75
Ohene et al. [30]	2013	Evaluation of antiretroviral therapy (ART) provision in an early cohort of patients initiating ART in Ghana	Eastern and Greater Accra region	Cross-sectional	Adults	683	Purposive	Self-report, (missed dose in the past 3 months)	78.6

### Adherence to antiretroviral therapy

**Fig 2:**
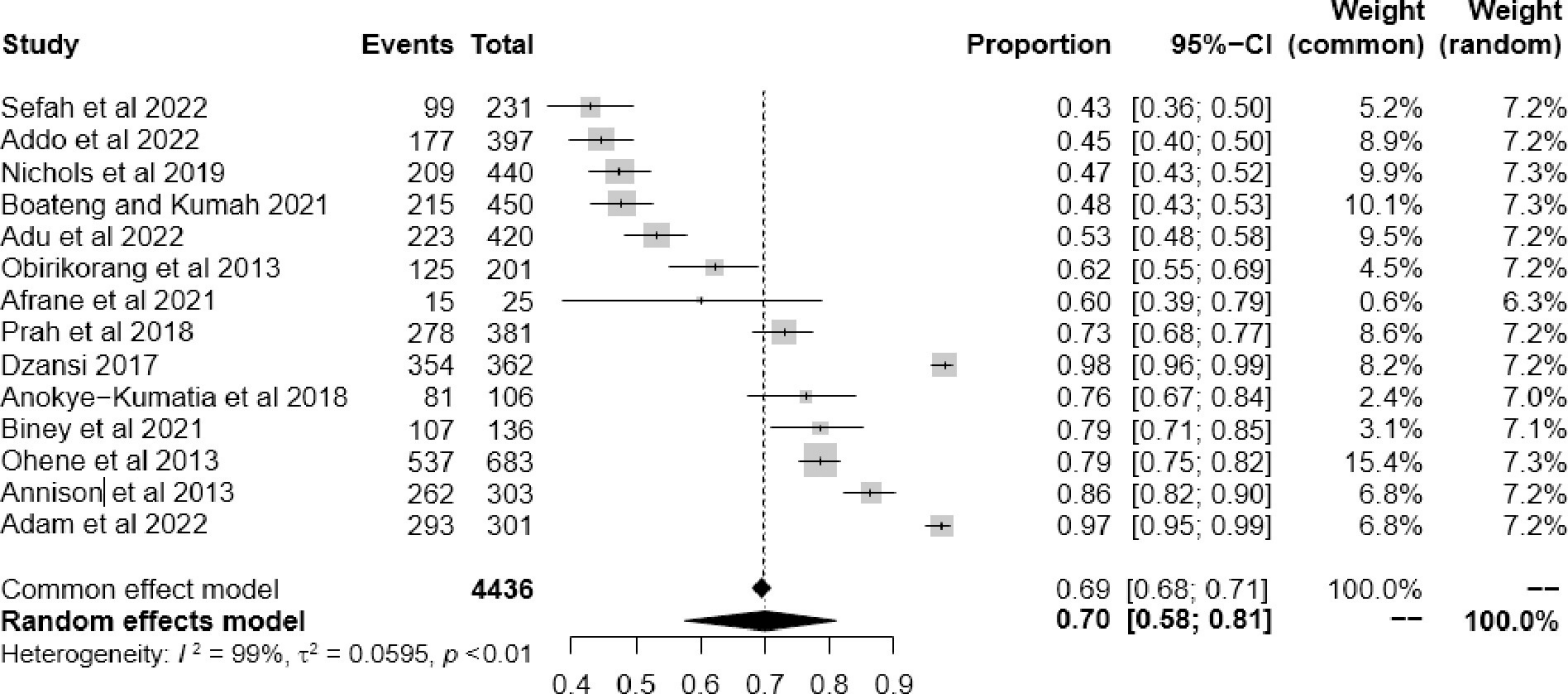
Forest plot of the pooled estimate of ART adherence among HIV/AIDS patients in Ghana.

The prevalence of adherence among the study participants ranged from 43% (95% CI; 36.0–50.0%) to 98% (95% CI; 96%-99.0%). The pooled estimate of adherence to ART was 70% (95% CI: 58.0–81.0) using a weighted-random effects model with significant heterogeneity (*I* ^2^ = 99%, p< 0.001) **[Fig 2].** This obvious heterogeneity was further supported by the drapery plot [51] for all the studies in the meta-analysis [**S1 Fig**]. In the drapery plot, the prediction region (light grey) is broader than the p-value curve of the pooled estimates, showing significant heterogeneity **[S1 Fig**].

### Sensitivity analysis

Sensitivity analysis was carried out to identify studies that contributed substantially to the pooled estimate and add to the overall heterogeneity of the meta-analysis. The baujat plot in **S2A Fig** shows that five studies—in the following order: Dzansi [50], Adam et al. [48] Nichols et al. [27] Addo et al. [23] and Boateng and Kumah [8] have a large impact on the pooled estimates and add considerably to the heterogeneity. Further sensitivity analysis was performed by omitting one study each at a time. As shown in **S2B Fig**, heterogeneity measured by *I*^2^ is ranked from low to high. The plot shows that omitting the study by Dzansi [50] and Adam et al. [48] resulted in *I*^2^ value of 98%. The weighted pooled adherence rate and heterogeneity *I*^2^ reduced after these five studies were excluded from the meta-analysis, falling to 69% (95% CI: 58.6–78.0) and 94% (p < 0.01), respectively.

### Subgroup analysis of the prevalence of adherence to ART among HIV patients

**Fig 3:**
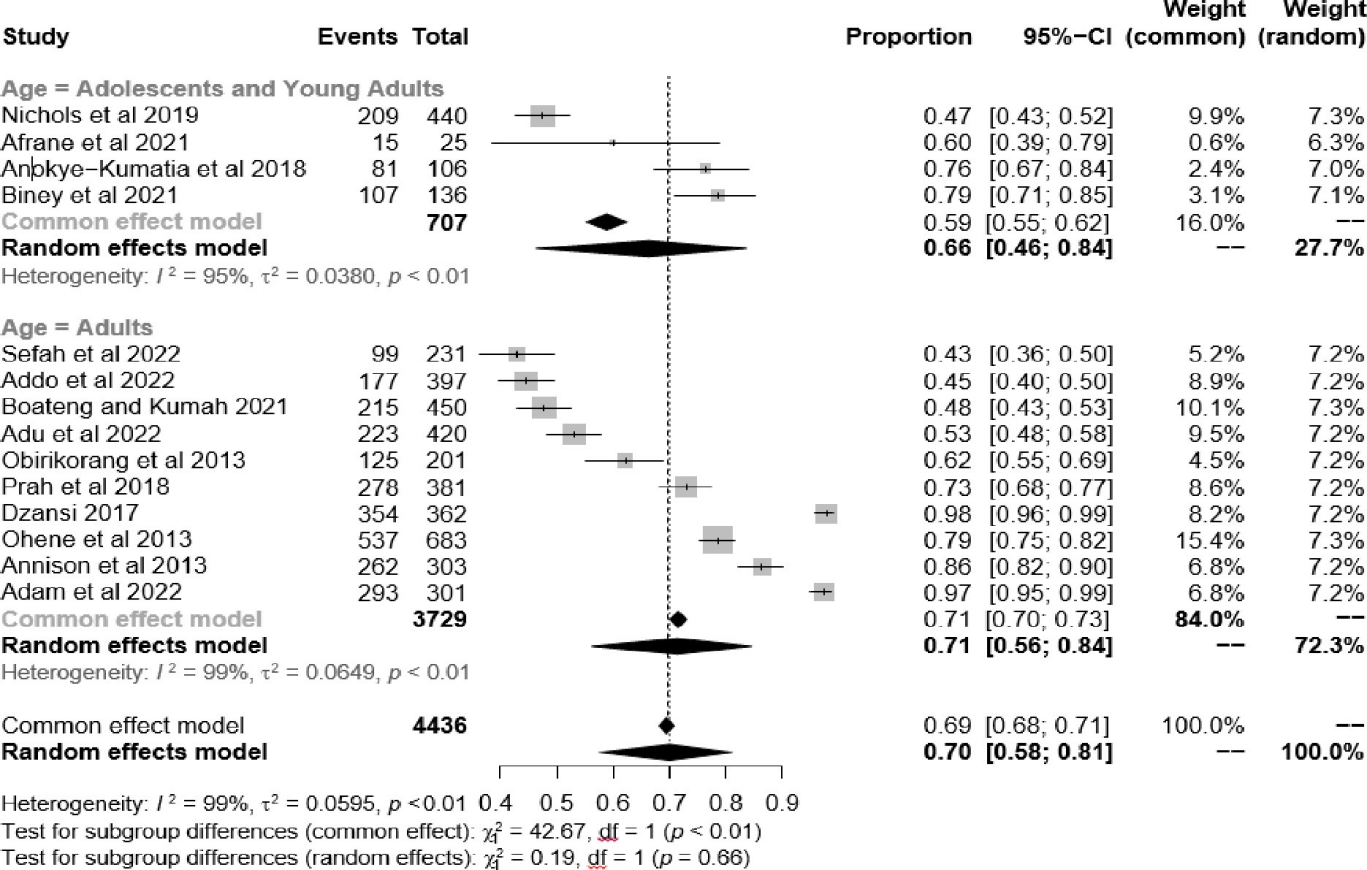
Forest plot showing age subgroup analysis of the prevalence of adherence to ART among HIV/AIDS patients in Ghana. The horizontal line represents 95% CI, and the diamonds correspond to the pooled estimate and CI. We used the random effect model.

Subgroup analyses were performed with respect to the age of study participants (adolescents and young adults, versus adults) to determine the prevalence of adherence rate. Findings showed that adolescents and young adults had the lowest adherence rate compared to adults [66% (95% CI: 46.0–84.0) vs. 71% (95% CI: 58.0–84.0), p = 0.66] [**Fig 3**]. Results of the meta-regression showed estimated regression weights for sample size and quality scores to be -0.0001 (p = 0.90) and -0.011 (p = 0.81), respectively. This means that for every unit increase in sample size and quality score, the pooled adherence of the studies is expected to decrease by 0.0001 and 0.011 respectively. The test of moderators for sample size (QM = 0.02; p = 0.90) and quality scores (QM = 0.06, p = 0.81) had no effect on adherence **(S3 Fig)**. This implies that neither sample size nor quality score could significantly predict the observed adherence estimate in the study.

### Quality of included studies

The mean quality score of the studies was 6.7 out of a maximum score of 8. The lowest and highest score was three and eight respectively. Of the studies that were included in the review; 11 [23–28,31,36,47,48,50], 2 [30,49], and 1 [46] were found to have a low, moderate, and high risk of bias respectively. Half of the studies, (50%) [23,27,28,30,46–48] scored “No” on question related to whether the outcome was measured in valid and reliable way. Three of the studies [25,46,49] did not clearly state criteria of inclusion of participants and were conducted in the Ashanti region with two employing convenience sampling [46,49] and the other random sampling [25].

### Publication bias

**Fig 4:**
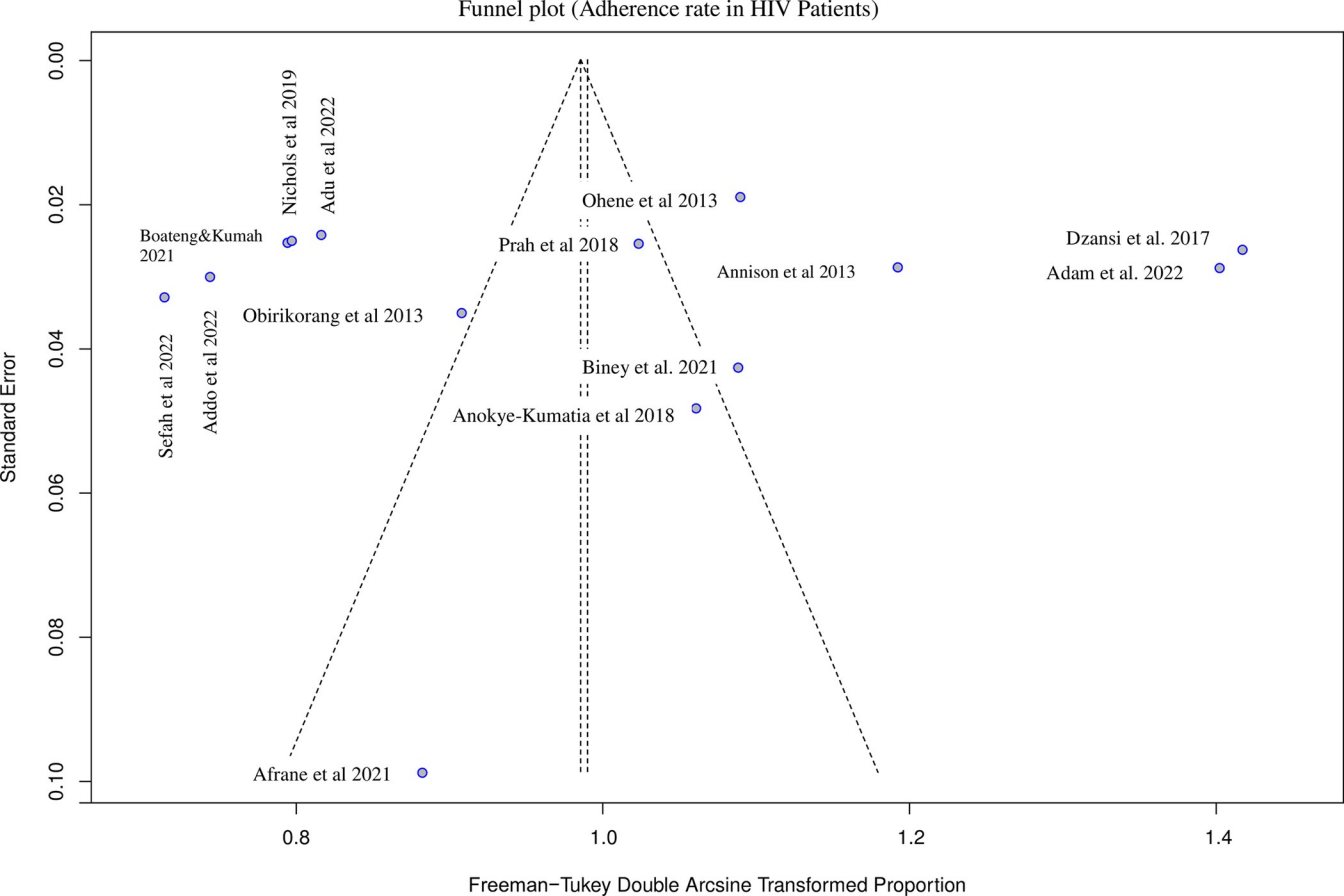
Assessment of publication bias. Begg’s funnel plot with a 95% confidence interval for adherence to ART.

The Begg and Egger funnel plot for prevalence of ART adherence showed a skewed pattern, with several studies at the top of the funnel plot, indicating the presence of studies with large sample sizes **[Fig 4**]. There was no publication bias as Egger’s test did not indicate the presence of funnel plot asymmetry (p = 0.86).

## Discussion

Adherence to medication remains a cornerstone to achieving better health outcomes among chronic diseases such as HIV/AIDS. Findings from this meta-analysis have shown a pooled adherence rate of 70% among HIV/AIDS patients in Ghana. This pooled adherence estimate is far below the recommended ≥95% required to achieve viral suppression [52–54]. This means critical attention and interventions are needed to prevent a higher likelihood of ART drug resistance, new transmissions, hospitalization, morbidity, and mortality among this population.

Our finding of 70% adherence rate is similar to the average adherence score of 72.9% reported by Heestermans et al. [55] in their review study in sub-Saharan Africa. However, the 70% adherence level in this study is slightly lower than the results reported by Mills et al. [21] in their review of published studies in Africa. Those authors reported a slightly higher adherence rate of 77% (95% CI, 68%-85%; I^2^, 98.4%) among HIV/AIDS patients taking ART in Africa. The findings of 70% adherence level is also far lower than the adherence estimate of 93.7% among HIV/AIDS children in Ethiopia [22]. The difference in these adherence rates could be due to the different methods used to assess adherence and the different populations studied. For instance, the study in Ethiopia focused on children less than 15 years whereas our study involved adolescents and the adult population [22]. These different population groups are likely to exhibit different medication adherence behaviors. The higher adherence rate in children could be due to the fact their parents provide support to adhere to their medication plan. Reporting bias and caregivers’ overestimation of self-reports [56,57] of adherence studies among children could lead to higher levels of adherence. Meeting the UN Sustainable development goal 3 of “ensuring good health for all at all ages” implies a needed focus on strategies and interventions for better health outcomes among HIV patients on anti-retroviral therapy.

In our sub-group analyses, adolescents and young adults had a lower adherence rate (66%) compared with adults (71%). This is similar to the studies by Kim et al. [58] where 62% of adolescents were reported to be adherent to ART. Several barriers have been identified related to ARV intake among young people living with HIV. Adolescents and youth often deny their HIV status, do not disclose their HIV status, and have the false perception that they feel better, leading to a lower perceived need for taking their medication [59–61]. They also feel stigmatized coming to the clinic for medication; this leads to frequent interruptions in adherence [62–64].

A common barrier to adherence to ART reported was forgetfulness. Forgetfulness is a well-documented barrier to adherence to ART [65–68] and several interventions including daily reminder messages have been implemented to address this barrier [69–72].

This study has both strengths and limitations. In terms of strengths, this is the first comprehensive review that has focused on the prevalence of adherence to ART among HIV patients in Ghana. A systematic search of the literature using strict inclusion criteria was conducted to ensure wider coverage and depth of the evidence that is available about ART adherence in Ghana. Furthermore, the methodological quality and strength of the evidence were critically appraised using tools recommended for critical appraisal of the literature. This makes our findings reliable and critical for use by policymakers to inform decisions. However, we acknowledge some limitations of the review. Of the 14 studies included in the review, two were not peer reviewed and this may raise concern about the quality of evidence produced as well as the credibility of the study outcomes reported. Furthermore, although we used a random effect model to account for the observed large heterogeneity from our meta-analysis, we could not significantly identify the potential source of the large heterogeneity in our meta-regression as only a few covariates were used. For example, covariates such as gender which has been reported to significantly explain the variation of adherence in similar studies [33] were not included in our data. Gender-specific information on adherence to ART was not reported in most of the included studies.

In resource-limited settings such as Ghana, self-report assessment of adherence is popular and widely used due to its ease of use in busy settings, affordability, and low staff requirements [73–75]. Over 60% of the included studies used self-report as opposed to objective measurements such as medication event monitoring system (MEMS), drug levels, or viral load outcomes to assess adherence. This method is subject to social desirability bias [76] and has been reported to overestimate adherence [74] and may affect the pooled adherence rate documented in this study. Notwithstanding these limitations, the findings of this meta-analysis can guide the research potential of strategic interventions to improve adherence to ART among PLHIV.

### Implications of findings

Findings from our review could be used by the Ghana National AIDS/STI Control programme (NACP) and the Ghana Health Service (GHS) to optimize ART clinical services and adherence support within the current healthcare systems. The sup-optimal ART adherence rate in this study implies a need for attention to design and implement integrated interventions (patient-friendly services, peer support, home visits, community support) and strategies to achieve viral suppression. The implementation of the interventions will also be important to reduce drug resistance, reduce new infections and reduce HIV-related morbidity and mortality among PLHIV, especially among adolescents and young adults who have increased risk of treatment failure due to sub-optimal adherence to ART. Healthcare professionals need to strengthen their capacity for counseling services during regular follow-up visits by PLHIV. There is also the need for implementation research that targets the several domains of the barriers to adherence to ART.

## Conclusion

A pooled adherence estimate of 70% was found among persons living with HIV/AIDS on antiretroviral therapy. Adherence was lower among young people compared with adults. A comprehensive approach in the form of a multi-component intervention in the domain of education, peer support, home visit, and daily reminder messages will be necessary to address the multi-dimensional complexities of adhering to ART for better treatment outcomes.

## Supporting information

S1 Fig Drapery plot showing p-value curves for adherence to ART among HIV/AIDS patients. Studies are represented by gray curves and weighted random effect models are represented on a grayscale. Studies with higher weights are shown in dark gray and those with low weights in light gray. Dashed horizontal lines are used to detect Confidence intervals for common alpha levels.
(DOCX)

S2 Fig Sensitivity analysis of the prevalence of adherence to ART. **(A**) Baujat plots to detect studies contributing to heterogeneity in the meta-analysis. **(B)** Plot for Leave-One-Out meta-analysis by omitting each study in turn.
(DOCX)

S3 Fig Meta-regression analysis plot to investigate the patterns of heterogeneity in our data based on sample size **(A)** and quality scores **(B)** of the studies.
(DOCX)
