# Supplementary material for: Adherence to antiretroviral therapy among HIV patients in Ghana: A systematic review and meta-analysis
Source: PLOS Glob Public Health. 2023 Nov 1;3(11):e0002448. doi: 10.1371/journal.pgph.0002448 (PMC10619784; doi:10.1371/journal.pgph.0002448)
Supplement: S1 Fig — Studies are represented by gray curves and weighted random effect models are represented on a grayscale. Studies with higher weights are shown in dark gray and those with low weights in light gray. Dashed horizontal lines are used to detect Confidence intervals for common alpha levels. (DOCX) [file pgph.0002448.s001.docx]

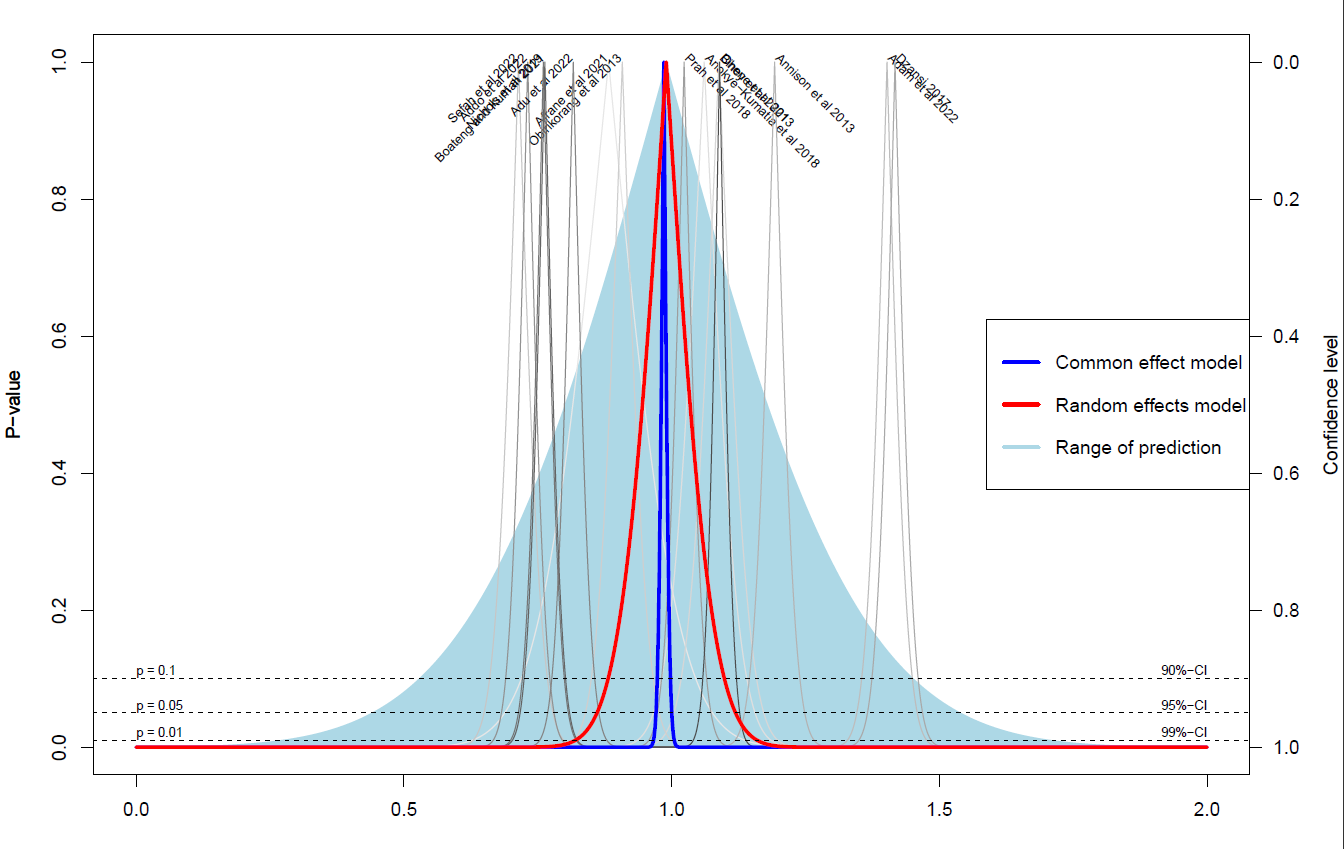


**S1 Fig:** Drapery plot showing p-value curves for adherence to ART among HIV/AIDS patients. Studies are represented by gray curves and weighted random effect models are represented on a grayscale. Studies with higher weights are shown in dark gray and those with low weights in light gray. Dashed horizontal lines are used to detect Confidence interval for common alpha levels.
