# Supplementary material for: Adherence to antiretroviral therapy among HIV patients in Ghana: A systematic review and meta-analysis
Source: PLOS Glob Public Health. 2023 Nov 1;3(11):e0002448. doi: 10.1371/journal.pgph.0002448 (PMC10619784; doi:10.1371/journal.pgph.0002448)
Supplement: S2 Fig — (A) Baujat plots to detect studies contributing to heterogeneity in the meta-analysis. (B) Plot for Leave-One-Out meta-analysis by omitting each study in turn. (DOCX) [file pgph.0002448.s002.docx]

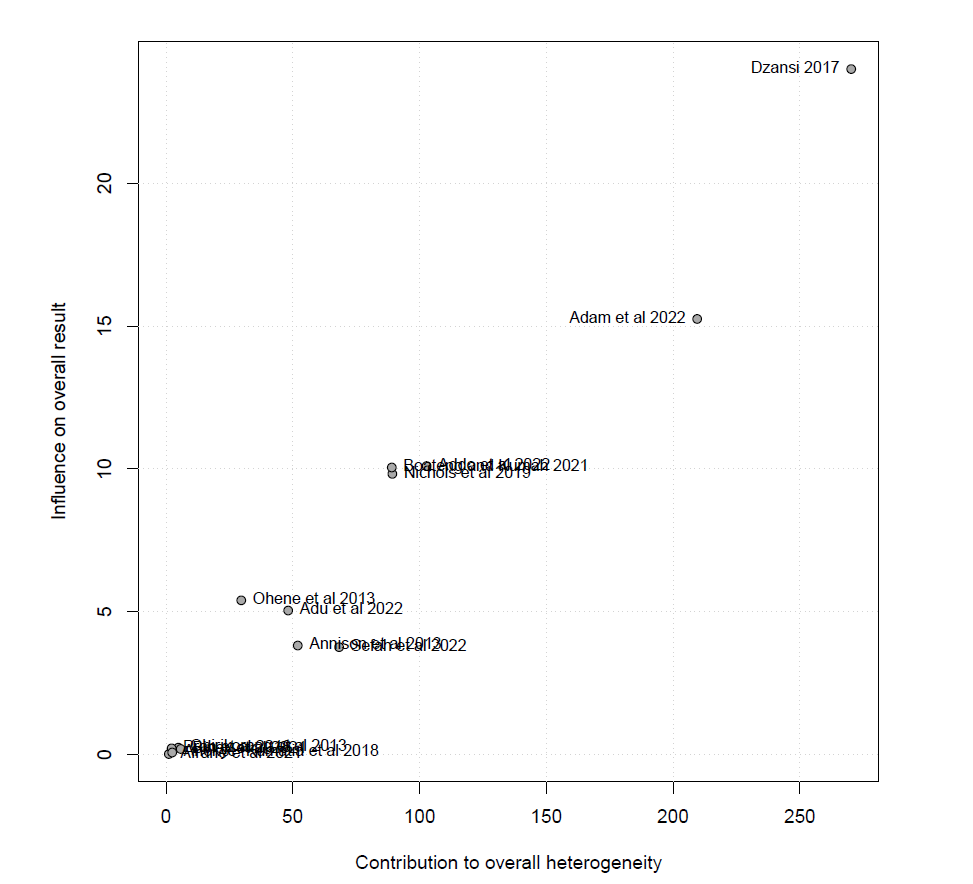

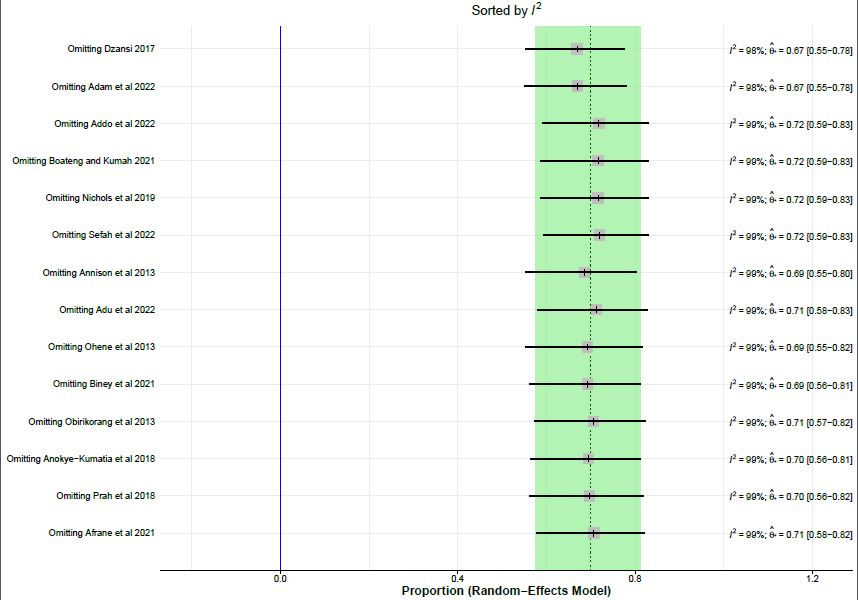


**a**

**b**

**S2 Fig:** Sensitivity analysis of the prevalence of adherence to ART. **(a**) Baujat plots to detect studies contributing to heterogeneity in the meta-analysis. **(b)** Plot for Leave-One-Out meta-analysis by omitting of each study in turn.
