# Supplementary material for: Adherence to antiretroviral therapy among HIV patients in Ghana: A systematic review and meta-analysis
Source: PLOS Glob Public Health. 2023 Nov 1;3(11):e0002448. doi: 10.1371/journal.pgph.0002448 (PMC10619784; doi:10.1371/journal.pgph.0002448)
Supplement: S3 Fig — Meta-regression analysis plot to investigate the patterns of heterogeneity in our data based on sample size (A) and quality scores (B) of the studies. (DOCX) [file pgph.0002448.s003.docx]

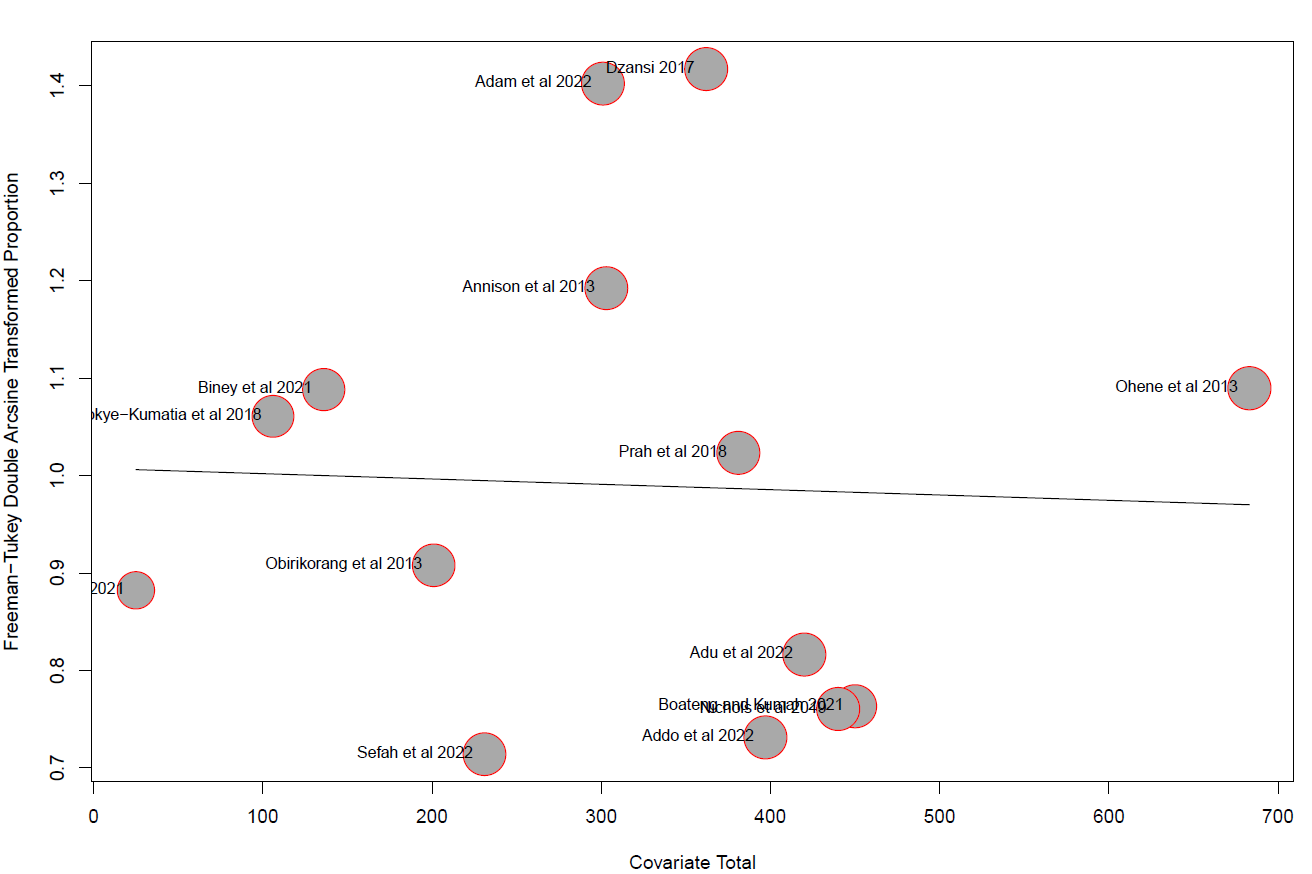


Estimate= -0.0001(95% CI-0.0009-0.0008)

QM (df=1) =0.0160, p-val=0.90


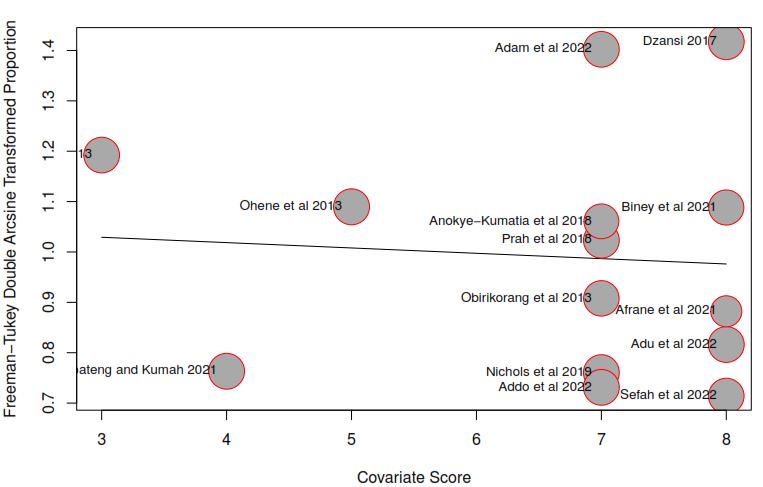


Estimate= -0.011 (95% CI-0.099-0.078)

QM (df = 1) = 0.0551, p-val = 0.81

**B**

**A**

**S3 Fig:** Meta-regression analysis plot to investigate the patterns of heterogeneity in our data based on sample size **(A)** and quality scores **(B)** of the studies
